# Supplementary material for: Endoscopic-Assisted Keyhole Middle Cranial Fossa Approach for Small Vestibular Schwannomas
Source: J Clin Med. 2022 Apr 21;11(9):2324. doi: 10.3390/jcm11092324 (PMC9101160; doi:10.3390/jcm11092324)
Supplement: Supplementary file 1 [file jcm-11-02324-s001.zip › jcm-1684911-supplementary.pdf]

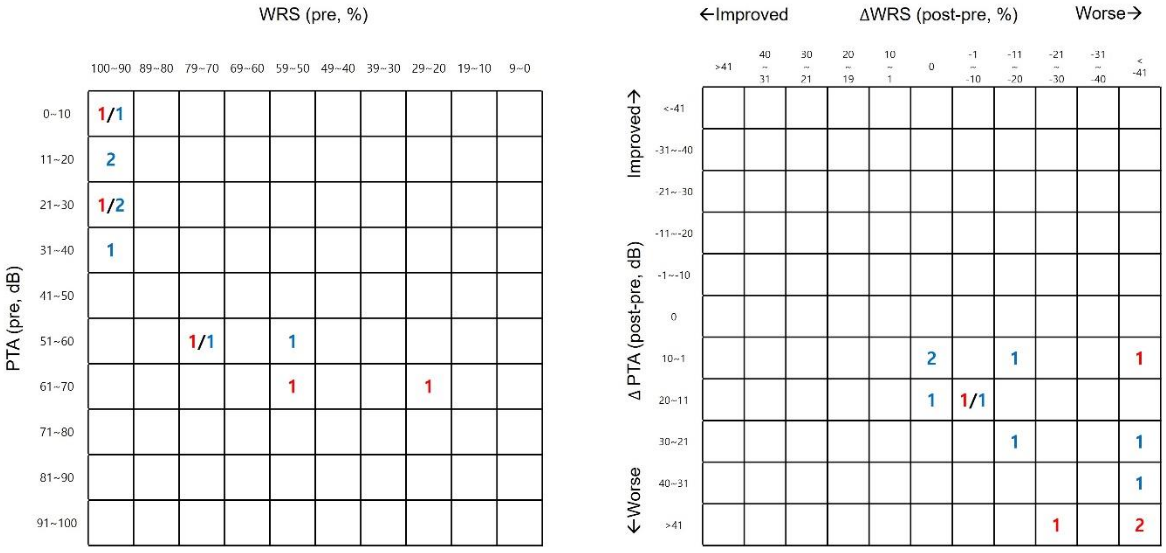

Supplementary Figure S1. Hearing outcomes of patients. Patients underwent VS removal via the keyhole middle cranial fossa approach (KMCFA, n=5, red) and the classical middle cranial fossa approach (MCFA, n=8, blue)

Left: Preoperative scattergram based on the 2012 American Academy of Otolaryngology-Head and Neck Surgery guidelines. X-axis indicates WRS, and Y-axis indicates PTA. The number of patients with preoperative audiologic data is depicted in the square. **Right**: Postoperative hearing change is shown. The deterioration of PTA and WRS after the surgery is calculated and depicted. The right lower quadrant includes the majority of patients. Even though there were no major complications after KMCFA, the outcome distribution was slightly different from that after classical MCFA, suggesting that the results were not promising.

WRS: word recognition score, PTA: pure tone average; VS: vestibular schwannoma
